# A *de novo* approach to disentangle partner identity and function in holobiont systems

**DOI:** 10.1186/s40168-018-0481-9

**Published:** 2018-06-09

**Authors:** Arnaud Meng, Camille Marchet, Erwan Corre, Pierre Peterlongo, Adriana Alberti, Corinne Da Silva, Patrick Wincker, Eric Pelletier, Ian Probert, Johan Decelle, Stéphane Le Crom, Fabrice Not, Lucie Bittner

**Affiliations:** 1 Sorbonne Université, Univ Antilles, CNRS, Evolution Paris Seine - Institut de Biologie Paris Seine (EPS - IBPS), F-75005 Paris, France; 2 Univ Rennes, CNRS, Inria, IRISA - UMR 6074, F-35000 Rennes, France; 3 Sorbonne Universités, CNRS - FR2424, ABiMS, Station biologique de Roscoff, Place Georges Teissier, 29680 Roscoff, France; 4 Institut de biologie François Jacob, GENOSCOPE, 2 rue Gaston Crémieux, 91057 Evry, France; 5 UMR8030, CNRS, Evry, France; 6 Sorbonne Université, CNRS - FR2424, Roscoff Culture Collection, Station Biologique de Roscoff, Place Georges Teissier, 29682 Roscoff, France; 7 Helmholtz Centre for Environmental Research – UFZ, Department of Isotope Biogeochemistry, Permoserstraße 15, 04318 Leipzig, Germany; 8 Sorbonne Université, CNRS - UMR7144 - Ecology of Marine Plankton Group, Station Biologique de Roscoff, Place Georges Teissier, 29680 Roscoff, France

**Keywords:** Holobiont, Meta-transcriptomic, *De novo* assembly, Marine, Plankton, k-mer based similarity

## Abstract

**Background:**

Study of meta-transcriptomic datasets involving non-model organisms represents bioinformatic challenges. The production of chimeric sequences and our inability to distinguish the taxonomic origins of the sequences produced are inherent and recurrent difficulties in *de novo* assembly analyses. As the study of holobiont meta-transcriptomes is affected by challenges invoked above, we propose an innovative bioinformatic approach to tackle such difficulties and tested it on marine models as a proof of concept.

**Results:**

We considered three holobiont models, of which two transcriptomes were previously published and a yet unpublished transcriptome, to analyze and sort their raw reads using Short Read Connector, a k-mer based similarity method. Before assembly, we thus defined four distinct categories for each holobiont meta-transcriptome: host reads, symbiont reads, shared reads, and unassigned reads. Afterwards, we observed that independent *de novo* assemblies for each category led to a diminution of the number of chimeras compared to classical assembly methods. Moreover, the separation of each partner’s transcriptome offered the independent and comparative exploration of their functional diversity in the holobiont. Finally, our strategy allowed to propose new functional annotations for two well-studied holobionts (a Cnidaria-Dinophyta, a Porifera-Bacteria) and a first meta-transcriptome from a planktonic Radiolaria-Dinophyta system forming widespread symbiotic association for which our knowledge is considerably limited.

**Conclusions:**

In contrast to classical assembly approaches, our bioinformatic strategy generates less *de novo* assembled chimera and allows biologists to study separately host and symbiont data from a holobiont mixture. The pre-assembly separation of reads using an efficient tool as Short Read Connector is an effective way to tackle meta-transcriptomic challenges and offers bright perpectives to study holobiont systems composed of either well-studied or poorly characterized symbiotic lineages and ultimately expand our knowledge about these associations.

**Electronic supplementary material:**

The online version of this article (10.1186/s40168-018-0481-9) contains supplementary material, which is available to authorized users.

## Background

In its scientific acceptation, symbiosis is defined as the living together of unlike organisms whatever the nature of their relationship [1], ranging from parasitism to mutualism. Symbiosis is a widespread phenomenon in the biosphere and plays crucial roles in evolution and ecology. One of the most popular examples of mutualism is the interaction between fungi and land plants, where fungi form mycorrhizae that help land plants to retrieve nutrients from soil [2]. In the ocean, benthic coastal ecosystems are structured and supported by symbiotic associations involving multipartners such as corals (Cnidaria, i.e., multicellular eukaryotes), microalgae (Dinophyceae, *Symbiodinium* spp., i.e. unicellular eukaryotes), and bacteria. Breakdown of this symbiosis ultimately leads to coral bleaching (the loss of photosynthetic symbionts), dramatically affecting the whole reef ecosystems [3]. While coral bleaching has been largely studied, there is a growing evidence that partners involved in this holobiont system contribute to make coral reef persistent in oligotrophic seas [4]. Symbiotic association between sponges (Porifera, i.e., multicellular eukaryotes) and bacteria (prokaryotes) allows bacteria to grow within the mesohyl matrix of the sponge where they can be metabolically active and persist in a highly oligotrophic habitat. The symbiotic interactions between sponges and bacteria are currently poorly understood from the genomic point of view [5]. Symbiotic associations involving two unicellular eukaryotes are also widespread in the oceanic plankton [5–8]. For instance, the cosmopolitan mutualistic associations between heterotroph Radiolaria (host) and endosymbiotic microalgae play significant ecological and biogeochemical roles in the oceans [9], but the underlying genomic basis of such associations remains uncharacterized. Although not cultivable in vitro, extraction of nucleic acids is nevertheless possible on such symbiotic partnerships, and this has recently allowed shedding light on the identity of the partners and their co-evolutionary history [6, 7]. Several symbiotic microalgae have been identified using such molecular approaches, and many of them belong to the Dinophyta [8]. However, mainly because of their highly complex and large genomes, the lack of reference genomes for both Dinophyta and Radiolaria make their study challenging for *de novo* assembly and functional annotation [10, 11].

**Fig. 1:**
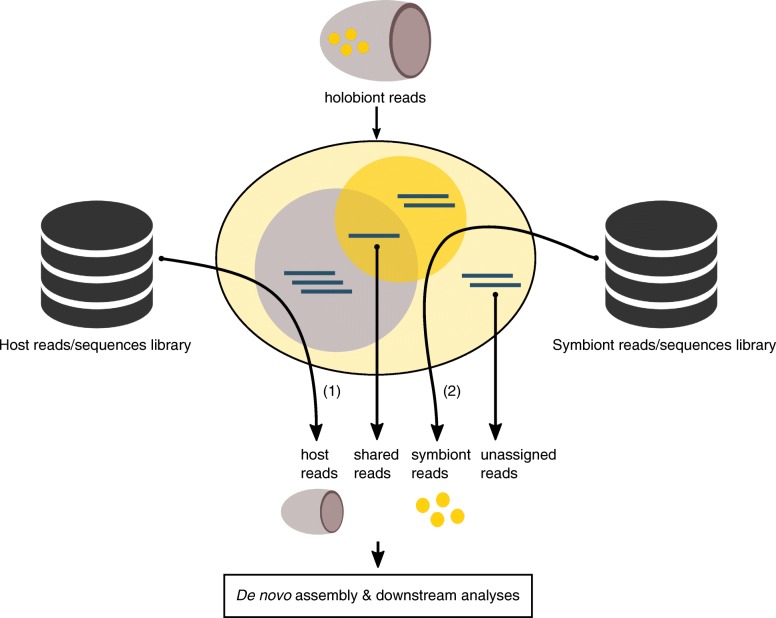
Theoretical overview on the application of SRC_c on a holobiont meta-transcriptome. The comparisons to (1) host and (2) symbiont reads/sequences library were done against the entire holobiont dataset to retrieve host and symbiont similar reads. The four resulting subsets (host, symbiont, shared, and unassigned reads) are then processed independently (*de novo* assembly and downstream analyses detailed in Material and Methods and in the Additional file 1).

Currently, RNA-seq is the best available approach to obtain large amount of genomic information from uncultured organisms isolated in the environment [12, 13]. RNA sequencing for a holobiont is now possible [14–16] and has promoted the development of sequencing projects [17] for non-model organisms. However non-model holobiont RNA-seq datasets correspond to a mixture of data coming simultaneously from the host and from the symbiont(s) (Fig. 1). Such datasets are a priori low complexity meta-transcriptomic datasets (i.e., that involve a reduce number of actors in comparison to soil, human gut, or marine microbial samples) and require *de novo* assembly of transcripts sequences, which implies large computational resources and introduces biases such as the creation of numerous chimeric sequences resulting from the misassembly of RNA fragments from the host and from the symbiont(s) [18, 19]. A variety of analysis strategies has been developed to address meta-transcriptomic challenges. Some of them avoid the assembly step to focus on identifying abundant species and significant functional differences between meta-transcriptomes directly from raw data mapping [20, 21]. Other strategies use statistical tools and machine learning algorithms to improve the quality of *de novo* meta-transcriptome assembly by learning from their abundance information [22].

**Fig. 2:**
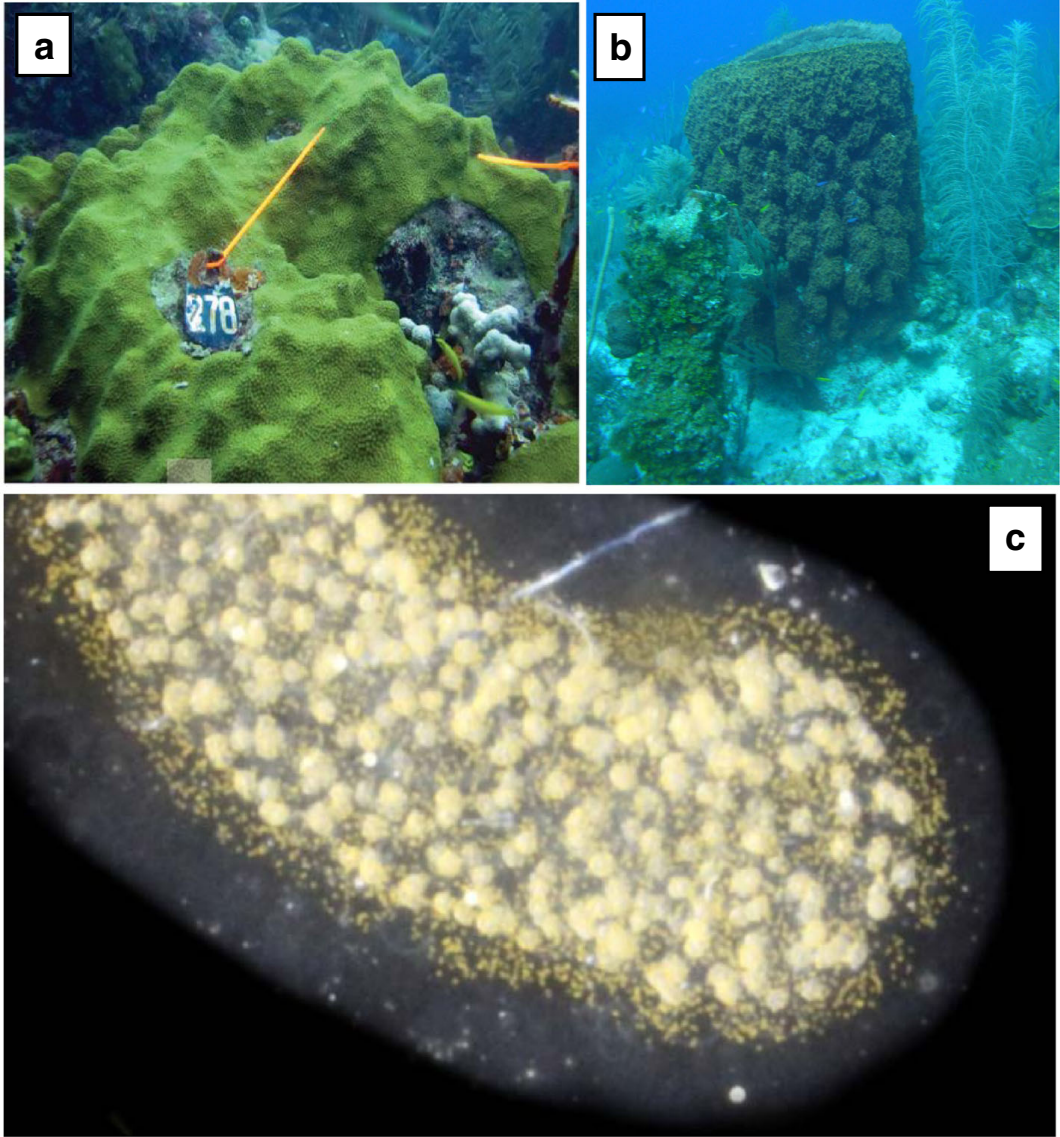
Pictures of the three holobiont models. **a** The *Orbicella faveolata* holobiont in symbiosis at reefs of La Parguera, Puerto Rico, in 2010 (credits: [24]). **b** A *Xestospongia muta* specimen in symbiosis on a coral reef near Little Cayman in the Caribbean (credits: Cara Fiore, January 14, 2015, http://feedthedatamonster.com). **c** A Collodaria colony with symbionts sampled in South Pacific Ocean at station 112.01 of the *Tara* Oceans expedition in 2011 (credits: Johan Decelle).

Here, we developed an original strategy aiming at improving the study of meta-transcriptomic datasets from holobionts. The concept relies on sorting the holobiont reads before the assembly step in order to distinguish the different actors (Fig. 1), and afterwards on processing independent *de novo* assemblies on each subset. To this end, we used a highly scalable tool, the Short Read Connector software in its Counter version (SRC_c) [23]. SRC_c is a fast kmer-based method initially developed to estimate the similarity between numerous (meta-)genomic datasets by extracting their common sequences. We focused on holobiont meta-transcriptomes for which a priori no or little genomic knowledge has been previously produced for host and symbionts, and we used SRC_c to compare these holobiont sequences to publicly available databases. We applied our strategy to disentangle the sequences and then *de novo* assemble the transcriptome of three distinct marine holobiont systems (Fig. 2). Two of them were already assembled and published and were used for qualitative comparison. The first model (M1) involves a Cnidaria host (*Orbicella faveolata*, belonging to the Metazoa) and Dinophyta symbionts (*Symbiodinium* spp., a unicellular eukaryote belonging to the Alveolata) forming a mutualistic association [3, 24] (Fig. 2a). This symbiotic association represents the best-known example of symbiosis in marine ecosystems, and many studies have been made trying to understand coral bleaching events (i.e., the loss of symbionts) [25, 26]. The coral holobiont also encompass other microorganisms consisting of bacteria, archaea, fungi, and viruses [27, 28]. In the second holobiont model (M2), the marine sponge *Xestospongia muta* (Porifera) harbors a dense (∼ 40% of its volume) and diverse microbial community including marine protists (e.g., fungi), archaea, and mainly bacteria [29–31] (Fig. 2b). The symbiotic associations between sponges and bacteria (suggested to be commensalism [32]) have become a major research focus to understand how sponges and their microbial communities can perform a variety of functional roles such as nutrition, cycling of metabolites, and host defense allowing them to proliferate in oligotrophic conditions [33, 34]. We chose a third, yet unpublished, holobiont dataset (M3) involving two distinct lineages of protists (unicellular eukaryotes): the radiolarian *Collozoum* sp. as host and Dinophyta symbionts belonging to the *Brandtodinium nutricula* species [6]. In this association, the radiolarian host forms a gelatinous matrix of several centimeters, which contains hundreds of host cells and thousands of symbiotic microalgae (Fig. 2c). Recent studies showed that this symbiosis is widely distributed in the ocean and significantly contributes to biomass and carbon export in the open ocean [35, 36].

As a proof of concept, we thus sorted the transcriptomic reads of these three holobiont models considering two major partners (i.e., using two reference libraries: one for the host and one for the symbiont), and then *de novo* assembled each of the subsets. We finally compared qualitatively and quantitatively the results (i.e., assembly metrics, functional and taxonomical annotations, presence of chimera) obtained when using SRC_c or not (Fig. 1; Additional file 1).

## Results

### Disentangling the holobiont sequences

To perform the sorting of the holobiont sequences for all three models (M1, M2, and M3), the SRC_c memory footprint was far lower than our cluster’s capacity (Table 1), even when indexing the biggest data set (i.e., the M2 symbiont library of 25 Gbp has been built with 58.9G of RAM). This reflects that further addition of data can be considered.

**Table 1:** Performances of SRC_c

		Time(hh:mm:ss)	Memory (Gb)
Cnidaria-Dinophyta holobiont (M1)	All symbionts library (M1a)	15:40:42	34.2
	*Symbiodinium* spp. library (M1b)	01:34:57	6.96
	Other symbionts library (M1c)	15:08:45	33.7
	Host library	01:06:56	3.9
Porifera-Bacteria holobiont (M2)	Symbionts library	21:04:47	58.9
	Host library	02:46:06	9.60
Radiolaria-Dinophyta holobiont (M3)	Symbionts library	07:05:28	4.10
	Host library	00:05:57	3.9

Memory peak and wallclock time of SRC_c indexing and query steps on the several data sets for models M1, M2, and M3

For the three holobiont models, the distribution within the four categories (i.e., host reads, symbiont reads, shared reads, and unassigned reads) obtained with the comparison of holobiont reads to reference host and symbiont sequence libraries is reported in Table 2.

**Table 2:** SRC_c assignment results for the holobiont models M1, M2, and M3

		# Reads	% Reads from holobiont
*Orbicella faveolata* holobiont (M1a)	Assigned to host library	498,008,661	64.26
	Assigned to symbiont library	56,011,798	7.23
	Shared	32,133,818	4.15
	Unassigned	188,870,747	24.37
	Total	775,025,024	
*Orbicella faveolata* holobiont (M1b)	Assigned to host library	500,145,229	64.53
	Assigned to symbiont library	54,850,148	7.08
	Shared	29,997,250	3.87
	Unassigned	190,032,397	24.52
*Orbicella faveolata* holobiont (M1c)	Assigned to host library	521,591,231	67.30
	Assigned to symbiont library	4,817,450	0.62
	Shared	8,551,248	1.10
	Unassigned	240,065,095	30.98
*Xestospongia muta* holobiont (M2)	Assigned to host library	6,193,678	19.04
	Assigned to symbiont library	825,154	10.64
	Shared	5,112,031	8.63
	Unassigned	21,090,174	61.69
	Total	33,220,038	
*Collozoum* sp. holobiont (M3)	Assigned to host library	3,188,944	3.26
	Assigned to symbiont library	23,234,402	23.72
	Shared	531,432	0.54
	Unassigned	71,003,016	72.48
	Total	97,957,794	

SRC_c assignment results for the Cnidaria-Dinophyta holobiont model (M1) against the complete Dinophyta library (M1a), the *Symbiodinium* spp. exclusive library (M1b), and the Dinophyta library excluding *Symbiodinium* spp. (M1c); the Porifera-Bacteria holobiont model (M2); and the Radiolaria-Dinophyta holobiont model (M3)

With M1, SRC_c assigned 64.3% of the holobiont reads to the cnidarian host and 7.2% to the Dinophyta symbiont full library (analysis M1a, Table 2). Restricting the symbiont library to the genus *Symbiodinium* spp. sequences allowed obtaining similar results with 64.5% of the reads identified as specific to the host library and 7.1% as specific to the symbiont library (analysis M1b, Table 2). On the contrary, when *Symbiodinium* spp. is removed from the library, only 0.6% of the holobiont reads could be assigned to the symbionts, and the proportion of reads assigned to the host increases up to 67.3% (analysis M1c, Table 2). Our tests on the symbionts library showed that the library content affected drastically the reads retrieval by SRC_c and demonstrated the sensitivity of the strategy. Considering these results, we focused on the M1a dataset for downstream analyses. We also noticed that shared reads (i.e., found in both host and symbiont libraries) always represent the lowest proportion of holobiont reads (M1a, M2, and M3).

### *De novo* assembly, contigs evaluation, and downstream analyses for M1 and M2

**Fig. 3:**
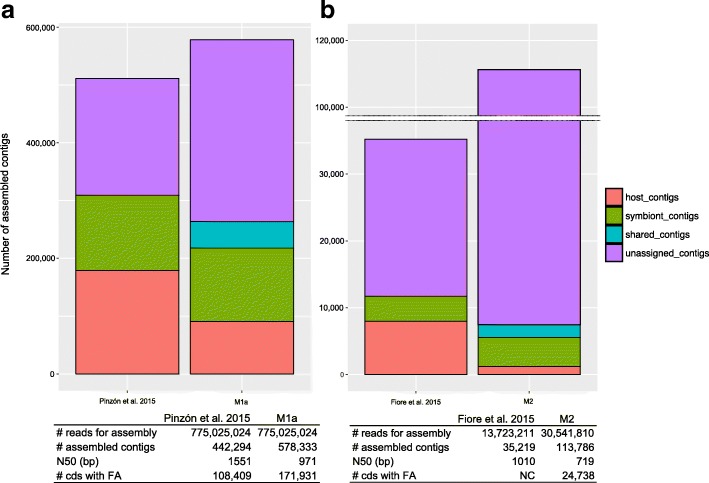
Metrics comparison between our results and the previous studies for the holobionts M1 (Cnidaria-Dinophyta) and M2 (Porifera-Bacteria). The total assembled contigs for holobionts M1a and M2 compared to the assembled meta-transcriptomes from **a** Pinzon et al. 2015 [24] and **b** Fiore et al. 2015 [30] are shown. General details about *de novo* assembly and functional annotation (termed FA) features are presented in corresponding tables for **a** holobiont M1a versus Pinzon et al. 2015 [24] meta-transcriptome, and **b** holobiont M2 versus Fiore et al. 2015 [30]. NC means that the exact number is not communicated.

For each holobiont meta-transcriptome, four subsets of reads were independently *de novo* assembled, producing contigs from which protein domains were then predicted and functionally annotated. For holobiont models M1a and M2, an overview of the analyses is available in the Additional file 1. The assembly metrics, statistics, and functional annotations from our contigs are summarized in Table 3. These metrics were directly compared to the one obtained in the original publications [24, 29] (Fig. 3). Compared to previous studies, it is worth noticing that we used a more up-to-date assembler [37] and a distinct annotation pipeline (cf. details in the “Methods” section). Reference databases for sequences annotation have also evolved since 2015, so the comparison of the quantitative values with previous studies are informative but have to be interpreted with caution. Our strategy allowed to obtain more assembled contigs (136,039 more contigs for M1a and 78,567 more contigs for M2), and the contigs metrics show shorter lengths of N50 (580 bp shorter for M1a and 219 bp shorter for M2) (Fig. 3). The M1a contigs display high remapping rates (> 80%) while M2 contigs show mixed results (25% < x < 86%) (Table 3). With M1a, a total of 255,223 protein coding domains were predicted for 44.1% of the assembled contigs, and functional annotations were found for nearly 30% of these protein coding domains (Table 3). With M2, protein coding domains were predicted for 39.6% of the contigs, and 54.9% of the domains were functionally annotated (Table 3). We obtained 1.6 times more functionally annotated contigs compared to [29] (M1a, Fig. 3). This comparison for M2 could not be made since the exact number of annotated contigs in the holobiont assembly has not been reported by the authors [24].

**Table 3:** *De novo* assembly metrics and downstream analysis of SRC_c resulting subsets for holobiont models M1a, M2 and M3

		# contigs	% contigs in holobiont	Smallest	Longest	N50	Mean length	% GC	Remapping rate (%)	# with ORFs	% of contigs with ORFs	Remapping rate of holobiont reads (%)	# predicted cds	% contigs with predicted cds	# annotated cds	% cds with functional annotations
Cnidaria-Dinophyta holobiont (M1a)	Host	90,558	15.66	201	29,214	1840	949	42	97.8	31,105	34.3	71.6	42,992	47.5	35,358	39
	Symbiont	127,212	22	201	13,093	1091	719	57	90.4	58,286	45.8	72.3	84,151	66.2	53,011	41.7
	Shared	46,017	7.96	201	7727	1067	796	55	82.3	28,075	61	41.4	38,547	83.8	25,382	55.2
	Unassigned	314,546	54.39	201	19,174	732	558	46	83.6	67,509	21.5	25.9	89,533	28.5	58,188	18.5
	Total	578,333								184,975			255,223		171,939	
Porifera-Bacteria holobiont (M2)	Host	2654	2.33	201	1921	299	311	42	44.4	215	8.1	17.6	707	26.6	593	83.9
	Symbiont	2431	2.14	201	5001	406	396	46	25	411	16.9	4.7	1072	44.1	988	92.2
	Shared	2324	2.04	201	751	301	299	54	86.4	8	0.3	22.3	163	7	30	18.4
	Unassigned	106,377	93.49	201	8811	748	572	39	73.2	29,520	27.8	59.1	43,150	40.6	23,127	53.6
	Total	113,786								30,154			45,092		24,738	54.9
Radiolaria-Dinophyta holobiont (M3)	Host	693	0.41	201	1209	277	303	42	65.2	44	6.3	10.6	123	17.7	49	7.1
	Symbiont	5207	3.08	201	1777	324	328	54	76.2	618	11.9	32	1468	28.2	942	18.1
	Shared	52	0.03	201	639	298	308	39	81.3	0	0	18.6	6	11.5	5	9.6
	Unassigned	162,947	96.48	201	10,569	714	580	41	89.7	49,032	30.1	73.2	72,420	44.4	44,772	27.5
	Total	168,899								49,694			74,017		45,768

To further test the usefulness of the reads sorting before the *de novo* assembly step, we compared the contigs assignment of M1a and M2 (column 1 in Table 3) with a usual taxonomic assignment performed with MEGAN6 [38] (Additional file 1). For M1a, MEGAN6 assigned 71,143 contigs to the host *Orbicella faveolata* and 148,409 contigs to the symbiont *Symbiodinium* spp. (Additional file 2). All the contigs assigned to *Orbicella faveolata* with MEGAN6 were also found with the SRC_c strategy (Table 3), but our method assigned 19,415 more contigs to the host category. On the contrary, MEGAN6 assigned 21,197 additional contigs to *Symbiodinium* spp. compared to our categorization strategy (Table 3, Additional file 2). With M2, MEGAN6 assigned 11 contigs to the host *Xestospongia muta* (Additional file 2) which is far less than the 2654 contigs defined with the SRC_c strategy (Table 3). However, MEGAN6 assigned also 33,810 contigs to *Amphimedon queenslandica*, a distinct sponge species which is not supposed to be the host in this holobiont system. MEGAN6 also succeeded to assign more contigs to bacteria (21,318 contigs) than the *SRC* strategy (2431 contigs) (Table 3).

Our functional annotations were compared to the one from the original studies [24, 29] (Additional file 1), but as previous publications do not provide exhaustive lists of the functional annotations and their corresponding abundance, these comparisons are essentially qualitative. Moreover, to minimize the biases, we focused on the more abundant annotations. For the *O. faveolata* host (M1), similarities were found in the top 15 of the most abundant annotations (Additional file 3). At the biological processes level, both our study and [24] found abundant metabolic process GO term (GO:0008152; 819 CDs (coding sequences) and 5278 genes, respectively). At the molecular function level, our host contigs mainly corresponded to binding protein (GO:0005515; 36,349 CDs) while authors of [24] mainly found catalytic activity functions (GO:0003824; 3361 genes). For M2, rare overlaps are found between the study in [29] and our annotations (Additional file 3): at the biological processes level, 1 of the top 15 host annotations is identical (signal transduction (GO:0007165)), and 3 of the top 15 symbiont annotations are in common (metabolic process (GO:0008152); proton transport (GO:0015992) and protein folding (GO:0006457)).

### Benchmark comparisons on M3: what difference does it make to use SRC_c?

For the holobiont model M3, we performed an entire assembly study of the yet unpublished meta-transcriptome, using the same assembly and annotation tools in order to compare, step by step, quantitatively and qualitatively the impact of using SRC_c (Additional file 1). In this way, assembly metrics, abundance of chimera, and functional contents were compared between the SRC_c contig sets (host, symbiont, shared, and unassigned) and a direct *de novo* assembled meta-transcriptome obtained from all holobiont reads (this strategy is hereafter called *noSRC*).

The assembly metrics appear very similar between *SRC* and *noSCR* (Table 4). A comparable number of reads were used for the assembly step, and a comparable number of assembled contigs were obtained. The N50 value for the *noSRC* strategy is slightly longer while the remapping rates are 5% better with the *SRC* strategy. Calculation times performed on the same bioinformatic cluster revealed that the *SRC* strategy was 40 h longer. The proportion of chimera detected with the *SRC* strategy fell to 0.247%, whereas it reached 0.465% without *SRC*. This reduction is clearly more significant in terms of number of sequences: 777 chimeras are detected without *SRC*, whereas 418 chimeras are detected with the *SRC* strategy. Most chimeras were contained in the unassigned set (Table 4). We noticed slightly less annotated CDs with the *SRC* strategy (45,768 against 47,260); however, the number and the composition in GO annotations were very similar (Additional file 4). We found 253 different biological processes with *SRC* against 255 with the *noSRC* strategy, and the top 5 functional annotations in the three gene ontology levels (Molecular Function, Biological Process, and Cellular Component) are strictly identical (Additional file 4). Considering all GO annotations, 686 are common to both strategies while 52 are exclusive to the *SRC* strategy and 42 to the *noSRC* strategy (Fig. 3, Additional file 4).

**Table 4:** SRC_c impact on Radiolaria-Dinophyta holobiont model (M3)

	no SRC	SRC
# reads used in assembly	48,733,956	48,660,697
# assembled contigs	167,023	168,899
# predicted cds	75,450	74,017
# annotated cds	47,260	45,768
N50 (bp)		
total	818	702
*host*		277
*symbiont*		324
*shared*		298
*unassigned*		714
remapping rates (%)		
total	85.6	90.5
*host*		65.2
*symbiont*		76.2
*shared*		81.3
*unassigned*		89.7
# chimera		
total	777	418
*host*		4
*symbiont*		47
*shared*		0
*unassigned*		367
Calculation time (min)		
total	330	2,783
*SRC*		2,460
*assembly*	330	323

*SRC* strategy’s impact on assembled contigs quality and calculation times of the Radiolaria-Dinophyta holobiont model (M3) compared to a direct meta-transcriptome assembly strategy (i.e., the *noSRC* strategy). In gray are displayed the details for the *SRC* strategy holobiont categories (host, symbiont, shared, and unassigned). The “total” values for N50 and remapping rates of the *SRC* strategy were re-calculated on pooled contigs from host, symbiont, shared, and unassigned subsets

To test the usefulness of the categorization step, the M3 contigs from the *noSRC* strategy were taxonomically assigned using MEGAN6 (Additional files 1 and 5). MEGAN6 assigned 10 contigs to Collodaria, whereas 693 contigs were assigned to the host category by the *SRC* strategy. MEGAN6 assigned 1383 contigs to Dinophyceae compared to the 5207 contigs categorized as symbionts by the *SRC* strategy. The leftover MEGAN6 contigs were assigned to bacteria and Archeae (3799 contigs), viruses (76 contigs), and other-eukaryotes (29,524 contigs), and 127,447 contigs remained unassigned (162,947 unassigned contigs with the categorization using the *SRC* strategy).

## Discussion

### The use of SRC_c to tackle meta-transcriptomic challenges

The strategy proposed here is a practical and scalable solution for transcriptomic assembly of non-model holobiont organisms, from which no or limited genomic information is available.

The present implementation of SRC_c [23] based on reference databases of putative partners involved in the holobiont consortium, and our analysis strategy, enabled the categorization of holobiont reads into four subsets. With respect to the reference libraries, as exemplified in M1, when the expected symbiotic partner (i.e., *Symbiodinium* spp.) is missing from the reference library, the number of reads assigned to the symbiont category decreases drastically from 50M reads to nearly 5M reads (Table 2). The M2 and M3 libraries do not contain reference data for the expected host partner, and consequently, only a low proportion of the holobiont reads are assigned to the host (19 and 3%, respectively). Accordingly, the proportion of unassigned reads is directly linked to both host and symbiont libraries content with respect to the studied holobiont. Overall, less unassigned reads were observed when the “correct” actors are involved (M1a: 24.4%) compared to the poorly studied models (M2: 61.6% and M3: 72.5%). These results highlight the sensitivity and specificity of the SRC_c requests that relies on the completeness of the database to accurately sort the reads of the holobiont. The SRC_c assignation step could be further improved by adding more sequences (i.e., reads, assembled genes, or transcripts) from taxonomically close species (from existing databases or newly produced) to the host and symbiont reference libraries, but also from multiple actors such as parasites and viruses that are common in multicellular and unicellular host cells. In this way, as SRC_c is a highly scalable tool, which has been improved since its first release [39] (Additional file 6), it is now possible and it will be relevant for future transcriptomic symbiosis studies to involve more than two reference libraries because symbiotic associations are often more intricate [27–31]. Involving more actors in the reference libraries will thus help to reduce step by step the proportion of unassigned holobiont reads.

We compared the SRC_c contigs metrics to those from previous studies (M1a and M2) [24, 29]. We found that not only our strategy allowed defining a new category of contigs (the “shared” contigs), but also allowed assembling more contigs than previous studies (Fig. 3). Our contigs metrics showed lower N50 for both models compared to previous studies but showed higher remapping rates overall for M1a (up to 90%, (Table 3)).

For M2, differences in the number of contigs as well as contigs metrics could be the results of the following: (i) the considered read set (we used the complete read set, whereas [29] used a reduced one, cf. details in the “Methods” section) and/or (ii) the use of distinct *de novo* assembly software (we used Trinity [37], whereas authors of [29] used the CLC workbench [CLC bio, Boston, MA, USA; (https://www.qiagenbioinformatics.com/)]. Previous studies had shown that Trinity is able to generate more assembled contigs than the CLC assembler when applied on the same dataset [40]. It is also known that assembled contigs from Trinity are shorter than those assembled by CLC but provided similar proportion of significant hits against the nr database [40].

With M1a, our strategy produced 1.5 times more CDs with a functional annotation (Fig. 3). At that point, we are unable to tell whether this observation can be the consequence of a better suited assembly strategy (SRC_c treatment and/or assembly software), and/or the use of a different annotation pipeline, and/or the supplementation of reference annotation databases between 2015 [24] and 2017.

With M3 analyses, we could estimate how SRC_c impacts the *de novo* assembly step and downstream analyses compared to a more conventional protocol (i.e., the *noSRC* strategy) (Table 4, Fig. 3, Additional file 1). The *SRC* strategy increased the total calculation time; however, compared to classic strategies, it allowed to create directly two subsets directly assigned to holobionts partners. Minimal differences were found between the *SRC* and *noSRC* strategies concerning the number of assembled contigs and, as for M1a and M2, the *SRC* strategy produced shorter contigs sequences with higher remapping rates. The *SRC* strategy helped to reduce importantly the number of potential chimeras. We conclude that the read sorting and assignation performed before the assembly step largely contributes to address one of the most delicate *de novo* assembly challenges [41]. Overall, the use of SRC_c for M3 might not be indeed so impressive in terms of metrics improvement, but it ensures the production of high-quality *de novo* assembled sequences (i.e., with high remapping rate and without chimera), which is crucial when studying non-model organisms, and which is a real gain for biologists who plan to perform molecular experiment based on these newly produced sequences.

### The SRC strategy offers new perspectives in functional annotations of holobiont partners

For all models, the *SRC* strategy led to a higher number of annotated contigs; however, as only partial information on the annotation content were provided separately for the host or the symbionts in previous publications [24, 29], we were mainly restricted to qualitative comparisons.

The comparison of the M1a host transcriptomes with the previous study meta-transcriptome [24] showed very few similarities for the most occurring functions, even if the most annotated function is common (i.e., metabolic process GO). Our 20 most occurring functions include signal transduction functions (14% of the total annotations) and molecule transport functions (8% of the total annotations) that do not appear in the most occurring function from [24]. These newly highlighted functions could help better understand the *Orbicella faveolata* host with respect to communication and cellular exchanges with its partners. We were not able to perform a similar analysis for the symbiont transcriptome since authors from [24] only focused on the host transcriptome. For M2, only 1/15 and 3/15 common annotations were found for host and symbiont, respectively. We suggest that the divergences in the analytical pipeline used, here Trinity versus CLC for *de novo* assembly followed by InterProScan versus FastAnnotator for functional annotation, make the functional annotation contents hardly comparable between studies. Despite these discrepancies, results from both analyses must be considered as potentially valuable and have to be checked with genome alignment when available or through in vitro validation when considering restricted group of functions (e.g., PCR).

Symbioses involving single cell heterotrophic hosts and photosynthetic symbionts have been described in the oceanic plankton using morphological and molecular data [5–7, 15]. Radiolarians and their symbiotic microalgae (e.g., Haptophytes, Dinoflagellates) have an ecological and biogeochemical significance [42–45], but little is known about symbiosis establishment and maintenance. If most microalgal symbionts can be grown in the laboratory as free-living stage [46], the study of radiolarian host only relies on single-cell isolation from the field [35, 47]. In this study, the radiolarian host belongs to the Collodaria order which is ubiquitous and abundant in the open ocean [35, 48]. Our knowledge about their ecology and evolution is limited, and hence, our analyses represent an opportunity to learn more about the genetic repertoire of such uncultivable, non-model lineage. Regarding functional annotations, the *SRC* and the *noSRC* strategies provided very similar results but the *SRC* strategy categorized the functional annotations among four subsets (host, symbiont, shared, and unassigned) (Additional file 5), which can be explored independently, allowing group-specific interpretations and biological hypothesis building for each partner from the holobiont. For instance, symbiont CDs linked to the photosystem I and II were detected, confirming that SRC_c succeeded to assign reads to photosynthetic actors, as expected here for the symbiotic partner (Additional file 4).

### Strategies regarding the use of SRC_c and future perspectives

SRC_c successfully compared different holobiont read sets to large reference libraries in less than 24 h, with reasonable computational resources (i.e., 10 CPUs and less than 20Go of RAM). By setting parameters (i.e., solidity threshold, k-mer size, similarity threshold), we adapted SRC_c to heterogeneous nature of sequences in libraries (i.e., length, raw reads or assembled genes/transcripts, data volume, k-mers distribution) and to poorly studied systems. When studying meta-transcriptome reads, selecting abundant k-mers helps to remove the one corresponding potentially to sequencing errors; however, rare sequence k-mers are consequently lost. On the contrary, when indexing already assembled sequences from genomes or transcriptomes, we do not expect a redundancy of the k-mers such as in high-throughput sequencing experiments, and we thus assume that any k-mer is relevant when it comes from a reference sequence.

Contrary to BLAST-like methods, SRC_c relies uniquely on shared k-mers for its similarity computation. It means that a certain amount of error-free k-mers (i.e., k-mers that do not contain sequencing errors) must be found in common in order to output sequences, which can make SRC_c less sensitive compared to alignment methods which authorize mismatches. However, contrary to alignment methods, SRC_c was tailored to scale to very high volume datasets, and comparisons presented in [23] showed that SRC_c could handle sets of orders of magnitudes higher volumes than BLAST (Additional file 6). SRC_c’s efficiency relies on its particular probabilistic data structure. The lightweight indexing and query of k-mers is made at the price of rare false positives. In our case, false positives correspond to k-mers that are not contained in the original indexed library. As in this work, the k-mer size was relatively low (i.e., 25), the default value for this parameter was kept ensuring a low false positives rate (Additional file 6). For longer k-mers (i.e., size > 31), we recommend to increase the size of the fingerprint if more precision is needed. SRC_c can also be used in a no-false positive mode that requires more memory, but that is still less costly than a hash table as demonstrated in [23].

In our tests, SRC_c helps to retrieve holobiont reads similar to host or symbiont close species. Previous tools like COMMET [49] already proposed such computation, although their data structure makes difficult the use of k-mers of small size, as computation time would be drastically impacted. SRC_c was thus chosen for its simple output and its adaptability to the heterogeneous nature of the libraries studied, notably by adapting the k-mer lowest occurrence and size parameters.

Future works on SRC_c parameters settings could include more extensive exploration of the impact of the similarity threshold parameter on the sensitivity of our approach. In this regard, if the reads similarity rate to the libraries could be relaxed, it may decrease the number of unassigned reads in particular for poorly studied models. A second strategy would be to implement an iterative enriching strategy to maximize the proportion of holobiont reads assigned to the host or to the symbiont. This strategy can allow to assign more sequences in the case of non-model organisms. After a first assignment round with SRC_c, holobiont reads linked to an identified group (host/symbiont) can be added to the reference libraries. Then, based on these new enriched libraries, a second run of SRC_c can be performed on the holobiont reads. This can be implemented as an iterative pipeline: at each round, more reads will be assigned to the host or symbiont categories and will then be used as reference libraries. Finally, the approach proposed here has been applied to holobiont systems (between two partners), but it could be used to address larger meta-transcriptomic datasets composed of more complex assemblages. Depending on the SRC_c library content, the user can choose to target either one or more specific species among the variety that composed such meta-transcriptomic datasets. Coupled to our assembly and downstream analysis strategy, the subsets resulting of the used of SRC_c are processed *de novo* allowing the potential discovery of newly assembled transcripts and the exploration of the functional and metabolic role for the first time of each partner without reference genome.

## Conclusions

SRC_c successfully processed a variety of large-scale datasets and offered a pragmatic way to classify sequences from different holobiont partners before assembly. We showed that our strategy allows improving assembly metrics in some cases and, in any cases, ensured to reduce the number of chimeras and to produce high-quality newly *de novo* assembled sequences. Our approach offers an efficient strategy to assemble and study holobionts involving non-model organisms. Overall, this *de novo* approach, allowing a taxonomic categorization of functionalities, can reveal the link between identity and function, which is necessary to better understand the functioning and contribution of each partner in holobiont systems. Applying our strategy will thus provide new insights into future adaptative and evolutionary studies of the symbioses.

## Methods

### Radiolaria-Dinophyta holobiont model (M3) sampling, RNA-seq library and sequencing

The Collodaria colony was sampled in the South Pacific Ocean at the station 112.01 (coordinates in decimal degrees: latitude − 23.3, longitude − 133.9) during the *Tara* Oceans expedition in 2011 [50]. The radiolarian colony of few centimeters in diameter was collected in situ at the subsurface (1 m deep) with a plastic jar, preventing disruption of the colony and aggregation of other planktonic organisms. Live observations through the binocular were performed to verify that no organisms were accidentally attached to the colony before preservation. The collected colony was directly isolated in 15 mL of RNAlater (ThermoFisher Scientific, Waltham, MA) and preserved at − 20 °C. Total RNA extraction was performed using NucleoSpin RNA kit (Macherey-Nagel, Düren, Germany) starting from a slice (about 1 cm diameter) of Collodaria PAC 37 colony. Briefly, frozen cells were transferred in a 1.5 mL tube containing 100 μL RA1 lysis buffer and grinded for 1 min with a motor driven pellet pestle previously refrigerated in liquid nitrogen. Then, 250 μL RA1 lysis buffer, previously mixed with 3.5 μL β-mercaptoethanol (1% of total RA1 volume), was added to the lysed cells, and the total volume was transferred to a Nucleospin filter. After centrifugation and addition of an equal volume of 70% ethanol, the RNA was purified following the manufacturer’s instructions and finally eluted in 40 μL nuclease-free water. Quantity and quality of extracted RNA were assessed by capillary electrophoresis on an Agilent Bioanalyzer (Agilent Technologies, Santa Clara, CA).

Finally, in order to reduce as far as possible the risk of residual genomic DNA, a further DNase treatment was applied on the total RNA using Turbo DNA-free kit (Thermo Fisher Scientific), according to the manufacturer’s protocol. After purification with the RNA Clean and Concentrator-5 kit (ZymoResearch, Irvine, CA), RNA was eluted in 10 μL nuclease-free water and used to synthetize cDNA with the Ovation RNA-seq System Version 2 (NuGEN, San Carlos, CA), following the manufacturer’s protocol. After cDNA shearing by Covaris E210 instrument (Covaris, Woburn, MA), Illumina library was prepared using the SPRIWorks Library Preparation System on a SPRI TE instrument (Beckmann Coulter Genomics, Danvers, MA), according to the manufacturer’s protocol without size selection. Ligation products were PCR-amplified using Illumina adapter-specific primers and Platinum Pfx DNA polymerase (ThermoFisher Scientific). After library profile analysis by Agilent 2100 Bioanalyzer and qPCR quantification (MxPro, Agilent Technologies), the library was sequenced using 101 base-length read chemistry in a paired-end flow cell on HiSeq2000 Illumina sequencer (Illumina, San Diego, CA), in order to obtain nearly 50 million paired end reads. Raw reads were deposited on the ENA database: https://www.ebi.ac.uk/ena/data/view/ERX2094044.

### Data retrieval and sequence libraries construction

For each of the three holobiont models (Fig. 2), we built reference sequences libraries representing host and symbiont(s) by selecting the taxonomically closest organisms available in public datasets (Additional file 7).

For the Cnidaria-Dinophyta holobiont model (M1), the host library includes 20 assembled transcriptomes (466,582 contigs) of cnidarian organisms [51] (including data from the host species *Orbicella faveolata* itself) and 2 genome-derived ESTs (201,677 ESTs) of *Nematostella vectensis* and *Orbicella faveolata* [52]. The symbiont library is composed of 123 RNA-seq reads datasets (a total of 5,563,498,607 reads) of Dinophyta (including the presumed major symbiont *Symbiodinium* spp. [53]) from the MMETSP project [54]. We built three versions of the symbiont reference library, one composed of all Dinophyta (M1a), the second exclusively composed of *Symbiodinium* spp. (15 RNA-seq datasets, a total of 123,122,726 reads) (M1b), and the third composed of all Dinophyta except *Symbiodinium* spp. (108 RNA-seq datasets, a total of 5,440,375,881 reads) (M1c).

For the Porifera-Bacteria holobiont model (M2), four RNA-seq datasets of poriferan species were included in the host library (642,229,924 total reads): *Amphimedon queenslandica* [55], *Crella elegans* [56], and both *Haliclona amboinensis* and *Haliclona tubifera* [57]. The M2 symbiont reference library corresponds to the *Tara* Oceans meta-genomic gene catalogue (OM-RGC) assembled from the pico-planktonic fractions (< 3 μm) including Eubacteria or Archaea [58]. It is composed of the bacterial gene catalog (40,154,822 assembled gene sequences) which has been downloaded from the OM-RGC website (http://ocean-microbiome.embl.de/companion.html).

For the Radiolaria-Dinophyta holobiont model (M3), we gathered Rhizaria sequences from four *de novo* assembled holobionts: 7215 presumed host transcripts were extracted among a total of 15,404 *de novo* assembled transcripts [15]. Host specific sequences were extracted from holobionts assemblies removing first sequences from prokaryotic origin with a BLASTn (e-value 1e-3) against the OM-RGC database, and second, removing symbionts sequences with a BLASTx (e-value 1e-3) against Dinophyta *de novo* assembled transcriptomes [46]. The exhaustive Dinophyta library created for the M1a was used for the reference symbiont library.

All reference libraries described above include assembled transcriptomes, genomes, or RNA-seq raw read datasets for eukaryotic or prokaryotic holobiont partners (Additional file 7). Their sizes vary from 4.5 Mbp to 25 Gbp with sequences length from 100 bp to 84 Kbp (Additional file 7).

### Comparing meta-transcriptomes (i.e., holobiont reads) to reference libraries using short read counter

#### Presentation of SRC_c

Short Read Connector Counter (SRC_c) [23] relies on a very lightweight data structure called a quasi-dictionary that enables to work with voluminous sequence sets. The quasi-dictionary enables to associate a piece of information to any element from a static set composed of N distinct elements. It is composed of two parts: a minimal perfect hash function (MPHF) [59] and a fingerprint table. The MPHF allows to index very efficiently the elements of the set in memory, such that each element can be associated to any piece of information (i.e., k-mer coverage, location in reads). The fingerprint table is used to verify the membership of an element to the indexed set of elements using the MPHF. This way, stranger elements to the MPHF can be filtered out. The quasi-dictionary is a probabilistic structure with a controlled false positive rate that depends on the size of the fingerprint. SRC_c needs as input two sets of sequences (that can be identical). To compare sequences from a query set Q to those from a target set T, the set indexed in the quasi-dictionary is a set of k-mers from T. Finally, for each sequence *S* from Q, the number of k-mers of *S* shared with T provides a similarity measure of *S* with the set T. This implies that the similarity measure given is asymmetrical: it depends on the placement of the k-mers on the reads of Q, not of those of T. SRC_c is available at https://github.com/GATB/short_read_connector, the commit 94aa6a65b5ddf61eba95108069fae29c41e51fb0 was used for this study.

#### Application on data

In this study, SRC_c is used to assign reads from a holobiont meta-transcriptome either to the host or to the symbionts. We divided the query of the holobiont dataset Q in two parts, one that consists in the comparison of Q reads to a bank (i.e., reference library) of host sequences and another that performs the comparison to a bank of symbiont sequences. The sets to index are composed of k-mers from the sequences. In each comparison, two sequence sets are considered: the whole holobiont set Q and the target bank set B. First, the set B, which contains reads or assembled sequences and represents sequences close to the host (resp. symbiont), is indexed. During the indexation phase, the solid set of k-mers (i.e., the set composed of any k-mer which occurrence is above a user-fixed threshold (the solidity threshold) in the data set) from T is computed using the DSK [60] method. This set is next indexed in the quasi-dictionary previously described. Then, the reads from the holobiont data set (Q) are queried. For each read, the query phase reports the abundance of its indexed k-mers. In the meantime, reads are checked to have enough positions (i.e., more than a given threshold which can be parameterized) for which an indexed k-mer starts over their length. This enables to add stringency to the query: a read that shares only a few k-mers with the index is considered not enough similar to the index. Finally, each read from Q (the holobiont) which was found similar to T (the host or the symbionts) during the query are returned in a binary vector and can be extracted to a FASTA format.

#### Parameters choice

Parameters from SRC_c were carefully chosen. First, the solidity k-mer solidity threshold was adapted according to the nature of the sequences in the bank data set. For libraries for which sequences were shorter than 300 bp with a relatively high coverage (e.g., M1 symbiont library involved only reads), the default value was kept (solidity threshold = 2). For longer sequences (e.g., M1 host library was composed of ESTs and M2 symbiont library was composed of *de novo* assembled genes), the threshold was adapted and set to 1. Due to the presence of small reads (50 bp) in our holobiont datasets, we also modified the default k-mer size value of 31 to a value of 25, so that any read contains at least a few k-mers. Usually, the k-mer size is higher [49]; however, 25 base pairs correspond to a decent value to ensure the uniqueness of the read [61]. During the query phase of SRC_c, a query sequence (from a dataset Q) must contain at least *s*% positions covered by at least one indexed k-mers (from a dataset B), to be considered similar to data from the set B [23]. As the *s* default value is set to 50%, it means that a read of size l should have at least l × *s* positions covered by (overlapping or non-overlapping) indexed k-mers. Consequently, when a large majority of the reads could not be assigned, our strategy was to decrease the *s* parameter from 50 to 40 in order to increase the quantity of recalled reads. We set the similarity value *s* to 50% for M1 and M3 and decreased it to 40% for M2. Both query and indexation phases are parallelized in SRC_c. For this study, analyses were performed on a Linux system with 40 cores, with the option -t 0 (maximal number of available threads is used), and 250 GB of memory.

### Read filtering, *de novo* assembly, and downstream analysis

For M1, M2, and M3 datasets, *S*ortMeRNA [62] has been used with default parameters with the Silva 104 SSU and LSU nr reference databases, in order to estimate the proportion of reads corresponding to rRNA sequences. For M2, in comparison to the original publication in which the CLC workbench has been used and 41% of rRNA reads has been detected [29], we finally chose to consider the total read set (i.e., 16,818,599 more reads than [29]) in the assembly step as the rRNA detection with SortMeRNA detected only 8% or rRNA reads.

All read subsets resulting from the SRC_c step were first filtered (sequences trimming and cleaning) with the Trimmomatic program [63] (v0.36) and custom parameter SLIDINGWINDOW:10:20. Filtered reads were assembled using the *de novo* transcriptome assembly program Trinity [37] (v2.4.0) with default parameters. The newly assembled contigs metrics were calculated with the Transrate program [64] (v1.0.3). Additional downstream analyses include protein coding domain prediction using Transdecoder [65] (v3.0.1) and functional annotation with InterProScan 5 [66] (v5.24-63), both with default parameters. The pipeline used for the steps described above is publicly available on a GitHub repository https://github.com/arnaudmeng/dntap [46].

### Taxonomic assignment with MEGAN6

The contigs sequences were compared to the nr database (August 2017 version) with the DIAMOND software [67] (v0.28.22.84) using default parameters for BLASTx comparison and a e-value of 1e-3. The resulting alignments were processed with the *daa2rma* tool script provided with MEGAN6, and GeneInfo Identifier (GI) was mapped to alignments using the gi_taxid.bin file (version of May 2017). Finally, taxonomic assignment has been calculated with default parameters using the MEGAN LCA (Last Common Ancestor) algorithm and was visualized through the MEGAN6 software.

### Chimera identification

We followed the protocol described in [68]. Fifty thousand randomly sampled *de novo* assembled contigs for the M3 (with the *SRC* strategy and without *SRC* strategy) were compared to the 7215 Rhizaria presumed contigs from [15] and 3,494,295 coding domains from *de novo* assembled contigs of 54 dinoflagellates transcriptomes [46]. The comparison was made using the BLASTx program [69] (e-value 1e-3). The tools scripts *detect_chimera_from_blastx.py* from [68] was applied to resulting alignments to detect potential chimeras.

## Additional files

Additional file 1: Detailed overview of the analysis strategy for M1a, M2, and M3. (PDF 45 kb)

Additional file 2: Taxonomic assignment of SRC assembled contigs with MEGAN6 for the holobiont models M1 and M2. (XLSX 23 kb)

Additional file 3: Details of common GO annotations M1 and M2 our contigs versus previous studies. (XLSX 38 kb)

Additional file 4: Comparison of functional annotations between *SRC* assembled transcriptomes and a *de novo* assembled transcriptome without the use of SRC_c in the case of holobiont model M3. Details of the functional annotations results for the *SRC* strategy applied to M3, the tables displayed correspond to the top 15 GO annotations found in host, symbiont, shared, and unassigned transcriptomes for the three levels of annotations (MF: Molecular Functions, BP: Biological Process and CC: Cellular Component). (XLSX 21 kb)

Additional file 5: Radiolaria-Dinophyta meta-transcriptome taxonomic assignment with MEGAN6. Table of taxonomic assignation of the 167,023 *de novo* assembled contigs from the assembly without SRC_c reads sorting of the holobiont model M3. (XLSX 9 kb)

Additional file 6: Details on the results and performances of SRC_c. (DOCX 15 kb)

Additional file 7: SRC_c library content information and data sources. Table with detailed information of SRC_c libraries contents. The type of data and the total library sizes are displayed. It includes taxonomic contents and links to data repositories for holobiont models M1, M2, and M3 and data that constitute SRC_c reads/sequences libraries. (XLSX 13 kb)
