# Supplementary material for: A de novo approach to disentangle partner identity and function in holobiont systems
Source: Microbiome. 2018 Jun 9;6:105. doi: 10.1186/s40168-018-0481-9 (PMC5994019; doi:10.1186/s40168-018-0481-9)
Supplement: Supplementary file 6 — Details on the results and performances of SRC_c. (DOCX 15 kb) [file 40168_2018_481_MOESM6_ESM.docx]

Additional file 6 Details on the results and performances of SRC_c:

*Software evolution and SRC’s improvements*

Short Read Connector (SRC) is a highly scalable and fast kmer-based method initially developed to estimate the similarity between numerous (meta-)genomic datasets by extracting their common sequences (Marchet et al, 2018 in press in Discrete Applied Mathematics, <https://doi.org/10.1016/j.dam.2018.03.035>). SRC is freely available since March 2017 on https://arxiv.org/abs/1703.00667 or <https://github.com/GATB/short_read_connector>. SRC has not been yet used to tackle concrete biological questions. Our study illustrates how the counter version of SRC (SRC_c) can be used in the context of a holobiont transcriptomic study. During our analyses, new features were thus added to the software such as the generation of specific binary format to store more efficiently SRC’s outputs, and the possibility to index only k-mers that belong both to the bank and the query in order to reduce the memory footprint.

*Illustration of SRC’s performances*

In its original publication [23], SRC was tested on data containing up to 8 billions k-mers (*i.e.* a *Tara* Oceans dataset corresponding to 189,207,003 Illumina reads, [http://www.ebi.ac.uk/ena/data/view/ERR599280SRC](http://www.ebi.ac.uk/ena/data/view/err599280src)). For this dataset, SRC took around 11 hours and used 110GB of RAM to compute the similarities of pairs of reads, while BLAST could process only a subsample of 1 million read in the timeout (200h CPU) set for the experiment.

*False positives of the method*

The data-structure of SRC has a probabilistic nature. The underlying hash function that is used to index k-mers is computed specifically for each data set that is needed to be indexed. This hash function is based on the set of k-mers present in the data. Then at the query, k-mers from a second data set are queried and their presence in the SRC’s index is checked. There are no false negative queries (if a k-mer is present, it will be detected).  However, if a k-mer that was not present in the original indexed set is queried, in rare cases SRC can wrongly interpret it as a present k-mer (false positive). If this happens, the number of shared k-mers between two reads can be artificially increased by this error. This can result a higher similarity score than the reality. Such events remain rare. Thus, the false positive rate of the method is theoretically assessed, and it was made possible to control it and make it as low as desired with the fingerprint parameter of SRC [23].
