# Supplementary material for: A de novo approach to disentangle partner identity and function in holobiont systems
Source: Microbiome. 2018 Jun 9;6:105. doi: 10.1186/s40168-018-0481-9 (PMC5994019; doi:10.1186/s40168-018-0481-9)
Supplement: Supplementary file 1 — Detailed overview of the analysis strategy for M1a, M2, and M3. (PDF 45 kb) [file 40168_2018_481_MOESM1_ESM.pdf]

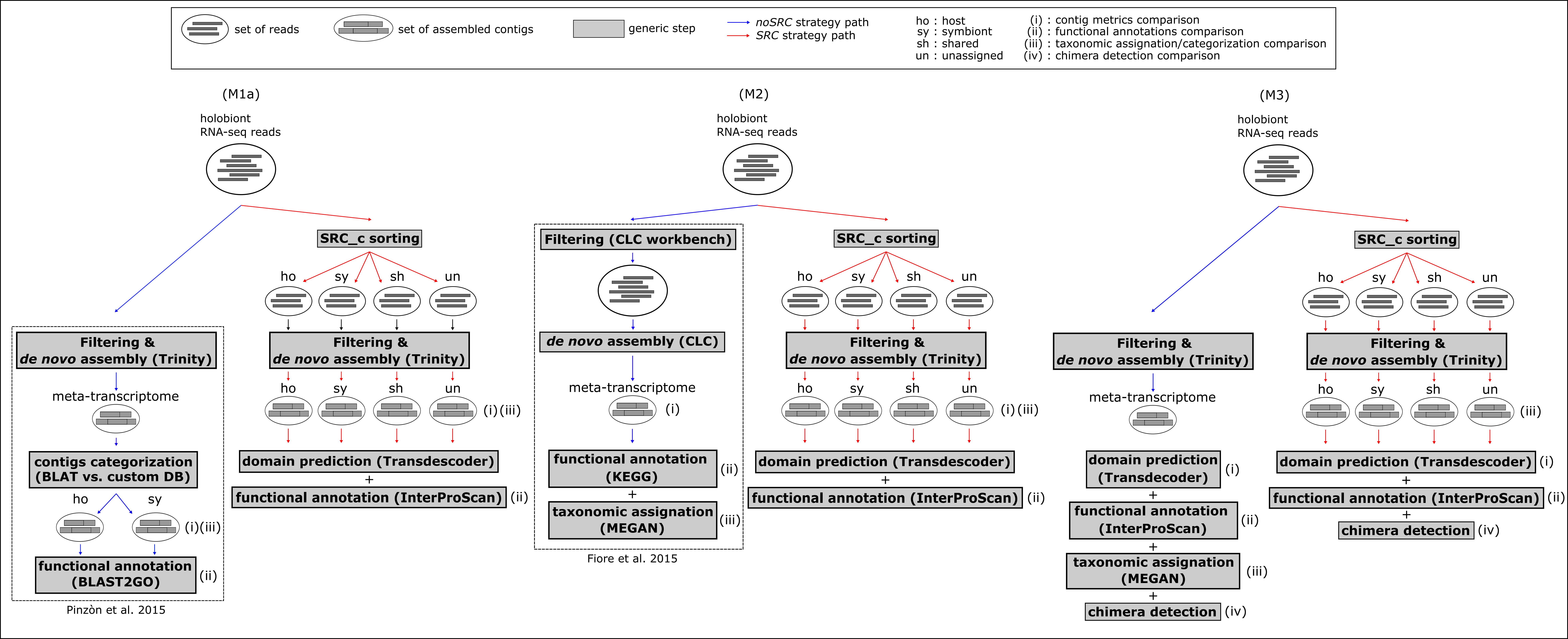

set of reads
set of assembled contigs
generic step
noSRC strategy path
SRC strategy path
ho : host
sy : symbiont
sh : shared
un : unassigned
(i) : contig metrics comparison
(ii) : functional annotations comparison
(iii) : taxonomic assignation/categorization comparison
(iv) : chimera detection comparison
(M1a)
holobiont
RNA-seq reads
Filtering &
de novo assembly (Trinity)
meta-transcriptome
contigs categorization
(BLAT vs. custom DB)
ho
sy
(i)(iii)
functional annotation
(BLAST2GO)
(ii)
Pinzòn et al. 2015
SRC_c sorting
ho
sy
sh
un
Filtering &
de novo assembly (Trinity)
ho
sy
sh
un
(i) (iii)
domain prediction (Transdescoder)
+
functional annotation (InterProScan)
(ii)
(M2)
holobiont
RNA-seq reads
Filtering (CLC workbench)
de novo assembly (CLC)
meta-transcriptome
(i)
functional annotation
(KEGG)
(ii)
+
taxonomic assignation
(MEGAN)
(iii)
Fiore et al. 2015
SRC_c sorting
ho
sy
sh
un
Filtering &
de novo assembly (Trinity)
ho
sy
sh
un
(i) (iii)
domain prediction (Transdescoder)
+
functional annotation (InterProScan)
(ii)
(M3)
holobiont
RNA-seq reads
Filtering &
de novo assembly (Trinity)
meta-transcriptome
domain prediction
(Transdecoder)
(i)
+
functional annotation
(InterProScan)
(ii)
+
taxonomic assignation
(MEGAN)
(iii)
+
chimera detection
(iv)
SRC_c sorting
ho
sy
sh
un
Filtering &
de novo assembly (Trinity)
ho
sy
sh
un
(iii)
domain prediction (Transdescoder)
(i)
+
functional annotation (InterProScan)
(ii)
+
chimera detection
(iv)
